# Downregulation of toll-like receptor 4 induces suppressive effects on hepatitis B virus-related hepatocellular carcinoma via *ERK1/2* signaling

**DOI:** 10.1186/s12885-015-1866-9

**Published:** 2015-10-30

**Authors:** Yiting Wang, Jing Cai, Xiaoli Zeng, Yajie Chen, Wei Yan, Yuming Ouyang, Dan Xiao, Zhiming Zeng, Long Huang, Anwen Liu

**Affiliations:** Department of Oncology, the Second Affiliated Hospital of Nanchang University, Nanchang, 330006 Jiangxi Province China

**Keywords:** HBV infection, HCC, Signaling pathways, TLR4, Tumor growth

## Abstract

**Background:**

Hepatitis B virus (HBV) infection is a major risk factor which can lead to development of hepatocellular carcinoma (HCC). In this study, we aimed to explore the effects of toll-like receptor 4 (TLR4) downregulation on the growth and survival of HBV-related HCC cells and to examine the molecular mechanisms been involved.

**Methods:**

The expression levels of TLR4 were examined in a panel of HCC cell lines (HepG2, SMMC7721, Huh7, HepG2.2.15 and Hep3B). The effects of TLR4 downregulation on the proliferation, apoptosis, and tumorigenicity of HBV-related HepG2.2.15 cells were determined. The effects of TLR4 downregulation on multiple signaling pathways were also measured. Co-immunoprecipitation and immunofluoresence staining assays were performed to investigate the interaction between TLR4 and HBV X protein (HBx).

**Results:**

The mRNA and protein levels of TLR4 were significantly increased in HepG2.2.15 cells than those in the other cells which have been studied. Downregulation of TLR4 significantly decreased the proliferation and induced G2/M cell cycle arrest and apoptosis in HepG2.2.15 cells. TLR4 depletion inhibited HepG2.2.15 cell colony formation and tumor growth in nude mice. TLR4 silencing decreased the phosphorylation of ERK1/2 but not JNK1/2, p38, or NF-κB. Chemical inhibition of *ERK1/2* approximately phenocopied the growth-suppressive effect of TLR4 downregulation on HepG2.2.15 cells. In addition, TLR4 showed a physical interaction with *HBx*.

**Conclusions:**

Taken together, TLR4 plays a tumor-promoting role in HBV-related HCC cells, which is associated with regulation of *ERK1/2* activation and interaction with *HBx*. Therefore, TLR4 may be a potential therapeutic target for HBV-related HCC.

## Background

Hepatocellular carcinoma (HCC) is the fifth most common cancer and the third leading cause of cancer-related deaths worldwide [1]. In 2012, it was estimated 554,000 new HCCs cases and 521,000 deaths from HCC in the world [2]. Chronic infection with hepatitis B virus (HBV) is accepted as a major risk factor which can lead to development of HCC [3]. Several HBV trans-activating factors such as HBV X protein (*HBx*), PreS2 activators, and hepatitis B spliced protein (*HBSP*) have been found to be implicated in hepatocarcinogenesis [4]. HBx protein can affect multiple cellular activities such as cell proliferation, cell cycle progression, and apoptosis [5], thus having a significant impact on the development of HBV-induced HCC.

Toll-like receptors (*TLRs*) are a family of transmembrane signaling receptors that are abundantly expressed on immune cells [6]. They can recognize a wide range of pathogens such as Epstein–Barr virus, HBV, and Helicobacter pylori, thus playing key roles in innate immunity [6–9]. In addition to immune cells, various types of tumor cells also express *TLRs* [10, 11]. It has been reported that TLR4 is overexpressed in HCC, compared to surrounding non-malignant liver tissues [12]. Compelling evidence indicates an important role for TLR4 signaling in hepatocellular tumorigenesis and progression [13, 14]. For instance, activation of TLR4 signaling by lipopolysaccharide (LPS) has been documented to promote cell survival and proliferation in HCC cells [14]. Stimulation of TLR4 usually leads to activation of multiple intracellular signaling pathways including nuclear factor kappaB (*NF-κB*) and mitogen-activated protein kinases (*MAPKs*) [15]. MAPKs phosphorylate specific serines and threonines of target protein substrates and regulate numerous biological activities such as gene expression, mitosis, apoptosis, and tumorigenesis [16].

Given that the importance of TLR4 signaling in tumor development and growth, in this study we aimed to explore the effects of TLR4 activation on the growth and survival of HBV-related HCC cells and to examine the molecular mechanisms involved.

## Methods

### Cell culture

HBV-unrelated HCC cell lines (HepG2, SMMC7721 and Huh7) and HBV-related HCC cell lines (HepG2.2.15 and Hep3B) were purchased from the Cell Bank of the Chinese Academy of Sciences (Shanghai, China). Cells were cultured in Dulbecco’s modified Eagle’s medium (DMEM, Solarbio, Shanghai, China) supplemented with penicillin (100 U/mL), streptomycin (0.1 mg/mL), and 10 % fetal bovine serum (Gibco, Grand Island, NY, USA) in a 5 % CO_2_ humidified incubator at 37 °C. G418 (6.5 mg/mL; Solarbio, Shanghai, China) was added to the culture medium to maintain HepG2.2.15 cells. LPS from Escherichia coli 0111:B4 was purchased from Sigma Aldrich (St. Louis, MO, USA). SB203580, SP600125, and PD184352 were purchased from Selleckchem (Houston, TX, USA) and pyrrolidine dithiocarbamate (PDTC) from Tocris (Bristol, UK).

### RNA interference

Small interfering RNAs (siRNAs) targeting TLR4 were designed and synthesized by Ribobio (Shanghai, China). Non-targeting siRNAs were used as negative controls (Ribobio, Shanghai, China). Individual siRNAs (50 nM) were transfected into cells with Lipofectamine™ 2000 Transfection Reagent (Invitrogen, MA, USA) according to the manufacturer’s instructions. After incubation for 24 and 36 h, transfected cells were collected for analysis of mRNA and protein expression, respectively.

### Western blot analysis

Whole cell lysates were prepared using lysis buffer [50 mmol/L Tris–HCl (pH 8.0), 150 mmol/L NaCl, 0.5 % NP-40, 0.1 % SDS, and 5 mmol/L EDTA (pH 8.0)] containing aprotinin (2 mg/L), phosphatase inhibitor Leupeptin (5 mg/L) and phenylmethylsulfonyl fluoride (PMSF; 1 mmol/L; Solarbio). Protein concentrations were determined using the BCA protein assay (Thermo Fisher Scientific, Rockford, USA). Equal amounts of proteins were subjected to 10 or 12 % SDS-polyacrylamide gel electrophoresis and transferred to polyvinylidene difluoride membranes (Millipore, Billerica, MA, USA). The membranes were blocked with 5 % fat-free milk and incubated with primary antibodies at 4 °C overnight, followed by incubation with secondary antibodies for 2 h. Signals were visualized by chemiluminescence (Thermo Fisher Scientific, Rockford, USA) and quantitated using Image J software. Primary antibodies used in this study were rabbit anti-TLR4 polyclonal antibody (1:1,500; Santa Cruz Biotechnology, CA, USA), rabbit anti-SAPK/JNK polyclonal antibody (1:1,000), rabbit anti-p44/42 MAPK (Erk1/2) polyclonal antibody (1:1,000), rabbit anti-Phospho-p44/42 MAPK (Erk1/2) (Thr202/Tyr204) polyclonal antibody(1:1, 000), rabbit anti-p38 MAP Kinase polyclonal antibody (1:1,000), rabbit anti-Phospho-p38 MAP Kinase (Thr180/Tyr182) polyclonal antibody (1:1,000), rabbit anti-NF-kB p65 polyclonal antibody (1:1,000), and rabbit anti-phospho-NF-kB p65 (Ser536) polyclonal antibody (1:1,000) (Cell Signaling Technology, USA), and rabbit anti-JNK1 + JNK2 (phospho T183 + Y185) polyclonal antibody(1:1,000) (abcam, USA), and mouse anti-β-actin polyclonal antibody (1:1,000) (Proteintech, Wuhan, China). Anti-rabbit (1:10,000) and anti-mouse (1:10,000) IgGs were purchased from Proteintech (Wuhan, China).

### Quantitative Real-Time Polymerase Chain Reaction (qPCR)

Total RNA was extracted using TRIzol (Invitrogen) and cDNA synthesis was performed using the PrimeScript RT reagent Kit (Takara, Japan). qPCR was performed using the SYBR Premix Ex Taq ™II (Takara) on an Applied Biosystems 7300 Real-Time PCR System (ABI, USA). The TLR4 primer sequence (PrimerBank ID: 373432602c1) came from PrimerBank (http://pga.mgh. harvard. edu/primerbank/): sense: 5′-AGA CCT GTC CCT GAA CCC TAT-3′; and anti-sense: 5′-CGA TGG ACT TCT AAA CCA GCC A-3′. The primers for amplification of *GAPDH* were as follows: sense: 5′-GTT GGA GGT CGG AGT CAA CGG A-3′; and anti-sense: 5′-GAG GGA TCT CGC TCC TGG AGG A-3′. All PCR amplifications were performed with an initial denaturation at 95 °C for 30 s, followed by 40 cycles of 95 °C for 5 s and 62 °C for 30 s.

### Cell counting kit-8 (CCK8) assay

Cells were seeded in 96-well plates at a density of 4000–5000 cells per well. Cells were allowed to adhere for 12 h and starved with serum-free medium for additional 12 h followed by treatment with 30 μmol/L of SB203580, SP600125, PD184352, or PDTC. The cells were exposed to 10 μg/mL LPS for different times. CCK8 (Zomanbio, China) was added to each well and incubated for 1 h at 37 °C. The optical density (OD) was measured at a wavelength of 450 nm using a microplate reader.

### Flow cytometry analysis

Cells were seeded at a density of 5 × 10^5^ cells per well in 6-well plates. After treatment, cells were fixed in ice-cold 70 % ethanol. Cell cycle distribution was analyzed using the Cell Cycle Analysis Kit (MultiSciences, China) and apoptosis was detected using the Annexin V-PE/7-AAD Apoptosis Detection Kit I (BD BioSciences, USA) according to the manufacturer’s instruction. Stained cells were examined by FACSCalibur flow cytometry.

### Tumorigenicity in nude mice

For tumorigenicity assays, 4- to 6-week-old male BALB/C nu/nu nude mice (16–18 g) were purchased from the Experimental Animal Center of Shanghai (Shanghai, China). Mice were randomly divided into 3 groups (*n* = 5 for each group) to receive a s. c. injection of 5 × 10^6^ HepG2.2.15 cells transduced with replication-defective lentivirus expressing *TLR4*-shRNA (Le-*TLR4*) or negative control shRNA (Le-NC) or normal saline (NS). Le-NC (2 × 10^7^TU, 40 μL/mouse), Le-*TLR4* (2 × 10^7^TU, 40 μL/mouse) or NS (40 μL/mouse) was injected intratumorally at several points every two days, with an accumulated dose of 1 × 10^8^ TU. Tumor volumes were measured every 5 days with a caliper and calculated according to the formula: 0.5 × length × width^2^. At 18 days after the cell inoculation, mice were sacrificed. The same treatments were done to other 3 groups of nude mice received HepG2 cells. All experimental manipulations were undertaken in accordance with the National Institutes of Health Guide for the Care and Use of Laboratory Animals, with the approval of the Scientific Investigation Board of the Nanchang University, Nanchang, China.

### Laser scan confocal microscopy

Cells were seeded on coverslips and allowed to adhere for 12 h. After treatment, cells were fixed with 100 % methanol for 30 min and blocked with 5 % bovine serum albumin (BSA) for 30 min. Coverslips were incubated with mouse anti-HBx monoclonal antibody (1:100, abcam) or rabbit anti-TLR4 polyclonal antibody (1:100, Santa Cruz Biotechnology) in 1 % BSA at 4 °C overnight, followed by with fluorescence labeled secondary antibody (1:100, Earthox, USA) in 1 % BSA for 30 min at room temperature. Cell nuclei were counterstained with 4′,6-diamidino-2-phenylindole (DAPI; Sigma). Images were captured using Nikon A1 Confocal Laser Microscope System and adjusted using NIS-Elements Viewer 4.0 (Nikon, Japan).

### Co-immunoprecipitation assay

HepG2.2.15 cells were grown on 10-cm cell dishes. After culture for 36 h, cells were harvested and lysed in the immunoprecipitation lysis buffer [20 mmol/L Tris–HCl (pH 7.5), 150 mmol/L NaCl, 1 % NP-40, 0.1 % SDS, 0.01 g/mL sodium deoxycholate, and 2 mmol/L EDTA(pH 8.0)] containing Aprotinin (2 mg/L), Leupeptin (5 mg/L) and PMSF (1 mmol/L). Total cell lysates were pre-cleaned with protein-A + G Sepharose Beads (7seabiotech, China) for 3 h at 4 °C and centrifuged. Aliquots of the supernatant were used as input. The remaining supernatants were incubated with mouse anti-HBx or anti-TLR4 antibody or isotype control IgGs and then with protein A + G Sepharose Beads (50 % slurry) at 4 °C overnight. After centrifuging, the pellets were resuspended with the SDS sample buffer and boiled to remove Sepharose beads. Lysate inputs and immunoprecipitates were then subjected to SDS-PAGE and analyzed by Western blotting. Total cell lysates were used as input control.

### Statistical analysis

Data were presented as mean and standard deviation (SD) for normally distribution. Groups were compared by one-way Analysis of variance (ANOVA) and multiple comparisons by LSD-*t* test using SPSS 21.0 (IBM SPSS for Windows, Version 21.0; IBM Corporation, Armonk, NY, USA). *P* < 0.05 was considered significant.

## Results

### TLR4 is overexpressed in HBV-related HepG2.2.15 cells

**Fig. 1:**
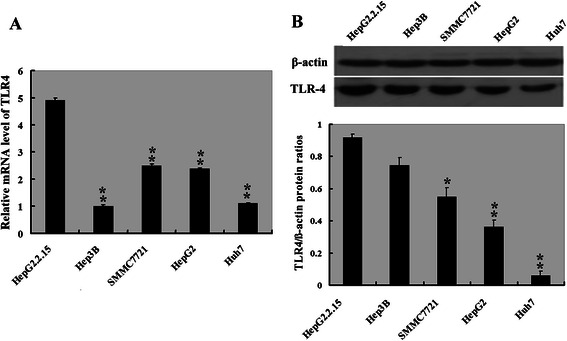
*TLR4* expression levels in five hepatoma cell lines. **a** Graph of relative ratios of mRNA of *TLR4* to *GAPDH* in each cell line. **b** Top: Protein expression of TLR4 was examined in HepG2, SMMC7721, Huh7, HepG2.2.15 and Hep3B by western blot; and bottom: Graph of relative ratios of protein of TLR4 to β-actin in each cell line. *P < 0.05, **P < 0.01, compared with HepG2.2.15, *n* = 3

We firstly examined the mRNA and protein expression of TLR4 in a panel of HCC cells (HepG2, SMMC7721, Huh7, HepG2.2.15 and Hep3B). As illustrated in Fig. 1a. the TLR4 mRNA level was significantly higher in HepG2.2.15 and SMMC7721 cells than in the other cell lines. However, the protein level of TLR4 was significantly greater in HepG2.2.15 and Hep3B cells than in HepG2, SMMC7721, and Huh7 cells (Fig. 1b).

### Targeted downregulation of TLR4 results in decreased growth and enhanced apoptosis of HepG2.2.15 cells in vitro

**Fig. 2:**
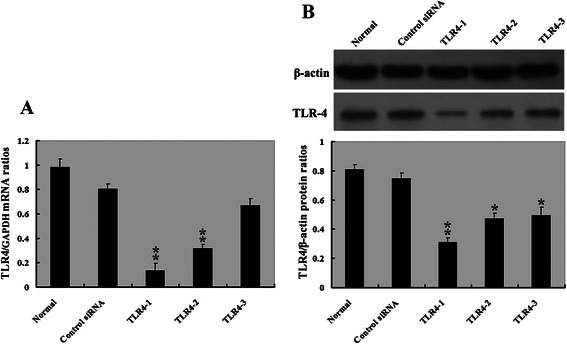
*TLR4* expression in untreated and treated group of HepG2.2.15. **a** Graph of relative ratios of mRNA of *TLR4* to *GAPDH* in each group. **b** Top: Protein expression of TLR4 was examined in Normal, Control siRNA, *TLR4-1*, *TLR4-2*, *TLR4-3* group by western blot; and bottom: Graph of relative ratios of protein of TLR4 to β-actin in each group. *P < 0.05, **P < 0.01, compared with Control siRNA group, *n* = 3

To explore the biological significance of TLR4 in HBV-related HCC cells, we specifically knocked down its expression in HepG2.2.15 cells using siRNA technology. This cell line was chosen because of its high expression of TLR4 both at the mRNA and protein levels. The amounts of TLR4 mRNA and protein were significantly reduced in cells transfected with one TLR4 siRNA (*TLR4-1*), showing efficient knockdown of TLR4 (Fig. 2). Similar results were observed in the cells transfected with another TLR4 siRNA (*TLR4-2*), with less effect of TLR4 downregulation. However, TLR4 expression levels were little affected by the transfection of the *TLR4-3* siRNA.

**Fig. 3:**
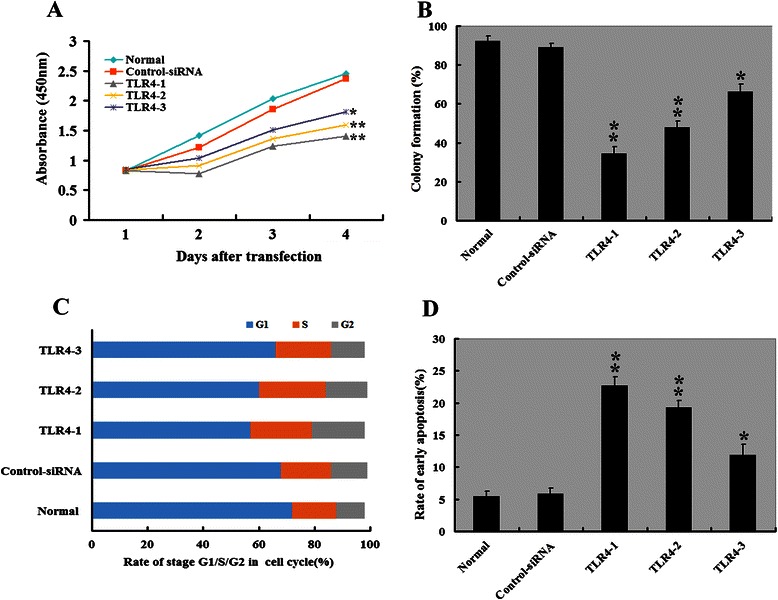
*TLR4* downregulation by siRNA-mediated silencing in HepG2.2.15 cells. **a** Cell proliferation was assessed by the CCK assay. HepG2.2.15 cells expressing *TLR4-1* or *TLR4-2* siRNAs showed a significantly reduced proliferation rate compared with those harboring *TLR4-3* or scrambled control siRNAs. **b** Colony numbers was assessed by colony formation assay. The quantitative analyses revealed HepG2.2.15 cells expressing indicated siRNAs showed a significantly reduced colony formation rate compared with those harboring normal or scrambled control siRNAs. **c** Cell cycle distribution was assessed by Cell Cycle Analysis Kit. HepG2.2.15 cells expressing indicated siRNAs showed a significantly increased rate in the G2 phase compared with those harboring normal or scrambled control siRNAs. **d** Early apoptosis was detected by flow cytometry. Early apoptotic cells were increased in HepG2.2.15 cells expressing indicated siRNAs compared with those harboring normal or scrambled control siRNAs. *P < 0.05, **P < 0.01, compared with Control siRNA group, *n* = 3

We next studied the impact of TLR4 silencing on HepG2.2.15 cell proliferation in vitro. CCK assay showed that downregulation of TLR4 by *TLR4-1* and *TLR4-2* significantly reduced the proliferation rate of HepG2.2.15 cells compared with control siRNA-transfected counterparts (*P* < 0.05; Fig. 3a). Colony formation assay further showed that TLR4 silencing resulted in a significant decrease in colony formation in HepG2.2.15 cells (*P* < 0.05; Fig. 3b). Cell cycle analysis revealed that TLR4 depletion caused a significant inhibition of cell cycle progression, leading to a selective accumulation of cells in the G2 phase compared with control siRNA transfectants (Fig. 3c). Quantification of apoptosis by Annexin V-PE/7-AAD double labeling indicated that TLR4-downregulated HepG2.2.15 cells had an about 3-4-fold increase in the apoptotic index, compared to control siRNA-transfected cells (Fig. 3d).

### Downregulation of TLR4 retards tumor growth in vivo

**Fig. 4:**
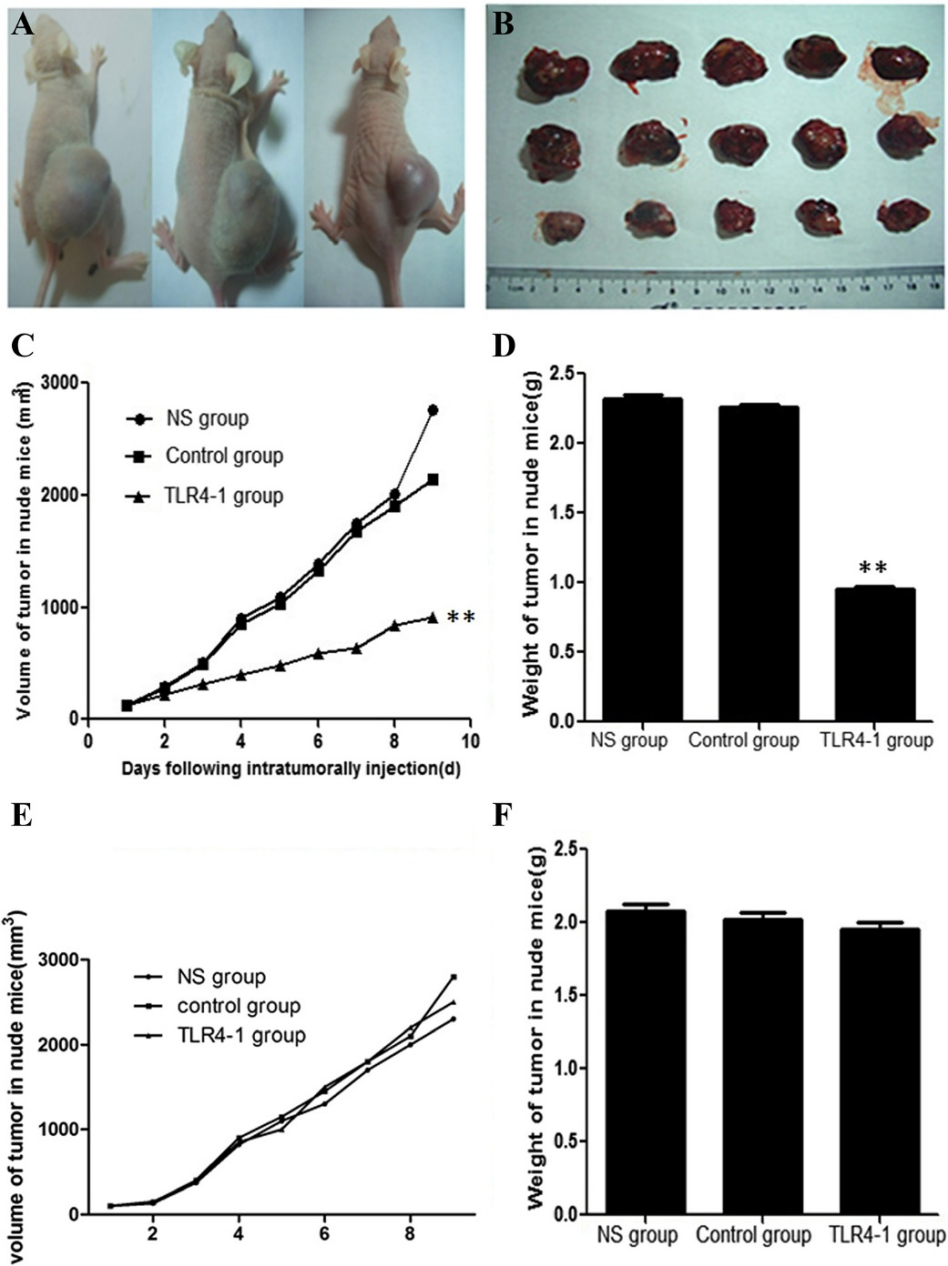
Downregulation of *TLR4* inhibits the growth of HepG2.2.15-derived xenografts in athymic nude mice. HepG2.2.15 transfectants expressing replication-defective recombinant lentiviral carrying the *TLR4*-shRNA(Le-*TLR4*) or replication-defective recombinant lentiviral carrying the negative control shRNA(Le-NC) were inoculated s.c. into immunodeficient mice. **a** Macrographic images of the HepG2.2.15 subcutaneous tumor xenografts in each mouse; **b** The size of subcutaneous tumor xenografts from each mouse; (*n* = 5 per group). **c** Growth curves of the tumor xenografts, volume of denuded tumor xenografts, volume of Le-*TLR4* group reduced significantly compared to control shRNA (Le-NC) group and normal saline group. **d** Weight of denuded tumor xenografts, weight of Le-*TLR4* group reduced significantly compared to control shRNA (Le-NC) group and normal saline group. **e** and **f** Volume and weight of tumor xenografts was not changed in nude mice received HepG2 cells **P < 0.01, compared with Le-NC group

To get more insight into the relevance of TLR4 silencing in vivo, an equal number of HepG2.2.15 cells infected with Le-*TLR4* or Le-NC recombinant lentivirus were injected s. c. into the right flank of nude mice. Compared to the NS group and control group, the Le-*TLR4* group showed a significant reduction in the tumor volume (*P* < 0.05; Fig. 4a-c). Simultaneously, the downregulation of TLR4 in Le-*TLR4* xenograft tumors contributed to a remarkable reduction in the tumor weight (*P* < 0.05; Fig. 4d). However, injection of HepG2 cells transfected with Le-*TLR4* or Le-NC recombinant lentivirus into nude mice did not inhibit the growth of tumor (Fig. 4e-f).

### TLR4 signaling regulates the activation of ERK1/2 in HepG2.2.15 cells

**Fig. 5:**
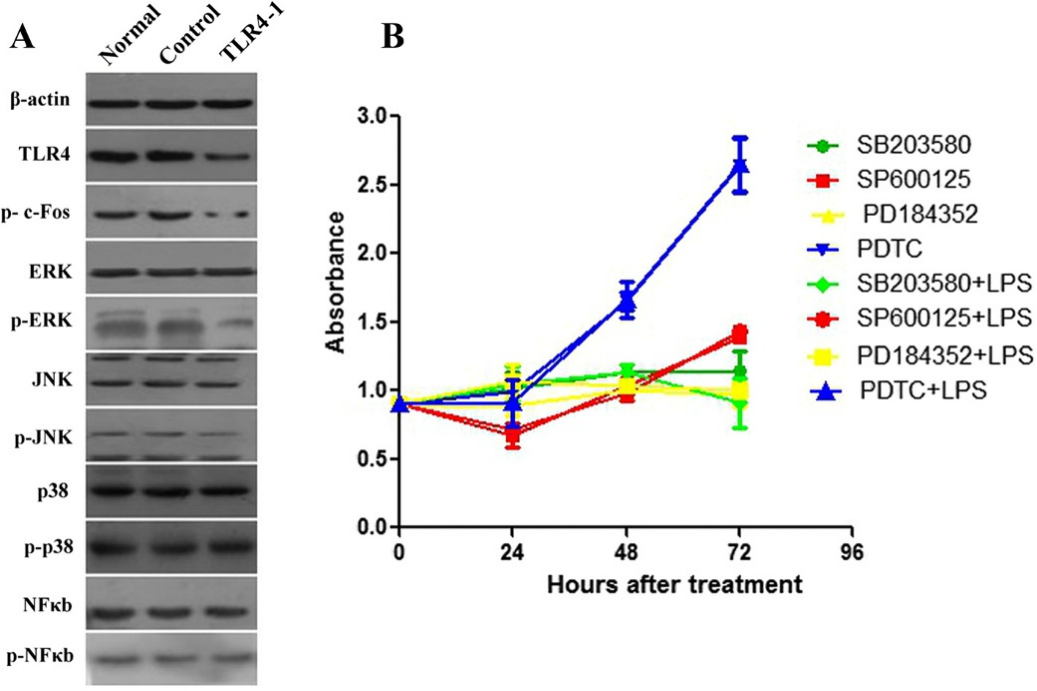
*TLR4* knockdown influences the expression and activation of multiple downstream genes. **a** Representative images of 3 independent experiments with similar results are shown, silencing of *TLR4* decreases the phosphorylation of ERK1/2 protein and c-Fos protein, a well-defined effector of *ERK1/2* signaling. However, silencing of *TLR4* has no impact on JNK1/2, NF-κB or p38 signaling. *P* < 0.05. **b** Chemical inhibitors SB203580, SP600125, PD184352 and PDTC were used to block *MAPKs* and *NF-κB* activation, and LPS was used to stimulate *TLR4* signaling. After indicated treatments, the cell viability was assessed at different time points using the CCK8 assay. Combined treatments with PD184352 and LPS significantly reduced the proliferation rate of HepG2.2.15 cells, compared with those treated with LPS and SB203580, SP600125 or PDTC. *P* < 0.05

To investigate the molecular mechanisms involved in the action of TLR4, we examined the activation of *ERK1/2*, *JNK1/2*, *p38*, and *NF-κB* by TLR4 silencing. As shown in Fig. 5a, ERK1/2 phosphorylation was remarkably disrupted in *TLR4-1* transfectants relative to control siRNA-transfected cells. In contrast, the phosphorylation levels of JNK1/2, p38, and NF-κB were comparable between *TLR4-1*-transfected and control siRNA-transfected cells. We also examined the effect of TLR4 downregulation on the phosphorylation of c-Fos, a well-defined effector of *ERK1/2* signaling. Notably, TLR4-downregulated cells showed a marked decline in phosphorylated c-Fos, compared to control siRNA-transfected cells (Fig. 5a).

To further confirm the mediating role of *ERK1/2* in TLR4 signaling, chemical inhibitors SB203580, SP600125, PD184352 and PDTC were used to block *MAPKs* and *NF-κB* activation, and LPS was used to stimulate TLR4 signaling. CCK assay showed that combined treatment with PD184352 and LPS significantly (*P* < 0.05) reduced the proliferation rate of HepG2.2.15 cells, compared with those treated with LPS and SB203580, SP600125 or PDTC (Fig. 5b).

### Interaction of TLR4 and HBx

**Fig. 6:**
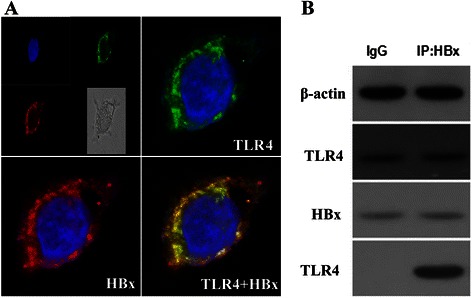
Structural and functional interaction of TLR4 and HBx in HepG2.2.15. **a** Immunofluorescence analysis revealed that TLR4 displayed a diffuse spotty distribution in the cytoplasm in HepG2.2.15 cells, and HBx had a similar cytoplasmic staining. **b** Lysates of HepG2.2.15 cells were immunoprecipitated with anti-HBx antibody and TLR4 protein was examined using Western blot analysis. TLR4 was detected in the immunoprecipitated HBx complex, but not in the IgG control sample. *P* < 0.05

Immunofluorescence analysis revealed that TLR4 displayed a diffuse spotty distribution in the cytoplasm in HepG2.2.15 cells, and HBx had a consistent cytoplasmic staining (Fig. 6a). To confirm the interaction between TLR4 and *HBx*, HBx was immunoprecipitated from HepG2.2.15 cells using anti-HBx monoclonal antibody and then subjected to Western blot analysis for the presence of TLR4. As shown in Fig. 6b, TLR4 was detected in the immunoprecipitated HBx complex, but not in the IgG control sample.

## Discussion

TLR4 is dysregulated in many human cancers, such as gastric cancer [17], colorectal cancer [18], and non-small cell lung cancer (NSCLC) [19]. Pathogenic stimulation is an important factor in modulating the expression of *TLRs* in cancer cells. For instance, Wang et al. [20] reported that Helicobacter pylori infection regulates TLR4 expression during gastric carcinogenesis. It has been documented that the expression of TLR4 is increased in HBV-related cirrhosis and HCC [21]. Wang et al. [22] showed that transfection of HK-2 cells, an immortalized proximal tubule epithelial cell line, with *HBx* gene results in upregulation of TLR4. Consistently, our data found that compared to the other HCC cell lines studied, HBV-related HepG2.2.15 cells had greater mRNA and protein levels of TLR4. These findings suggest that TLR4 expression is regulated in HCC cells at least partially by *HBV* infection, largely at the transcriptional level.

Accumulating evidence indicates that dysregulation of TLR4 is causally linked to tumor development and growth. TLR4 silencing has been found to suppress human NSCLC cell growth [19]. Yuan et al. [23] reported that activation of TLR4 signaling promotes gastric cancer progression by inducing the production of mitochondrial reactive oxygen species and subsequent activation of Akt and NF-κB signaling [23]. In contrast, constitutively active TLR4 was found to reduce tumor load in an APC (Min/+) mouse model of colorectal carcinoma through induction of apoptosis [24]. These studies suggest that TLR4 plays a complex role in tumor development and progression. Our data revealed that TLR4 downregulation decreased the proliferation and induced cell cycle arrest and apoptosis in HepG2.2.15 cells, indicating its requirement for HBV-related HCC cell survival and growth. In vivo studies confirmed the mediating role of TLR4 in HBV-related HCC tumor growth. TLR4 downregulation may have a specific role in limiting the progression of HBV-related tumor, while HBV-unrelated HepG2 cells cause tumor development in nude mice with or without TLR4 downregulation. In agreement with our findings, Dapito et al. [25] demonstrated that TLR4 signaling is implicated in HCC progression, mediating increased proliferation and prevention of apoptosis.

HBx plays a critical role in HBV-related HCC pathogenesis [5]. Our biochemical studies revealed a physical interaction between TLR4 and HBx in HepG2.2.15 cells, which may account for the tumor-promoting effects of TLR4 in HBV-related HCC cells. HBx protein is capable of regulating several intracellular signaling pathways, such as NF-κB and MAPKs [26, 27]. Interestingly, we found that TLR4 downregulation interfered with the activation of ERK1/2, but not JNK1/2, p38, or NF-κB. Moreover, the effect of TLR4 silencing o HepG2.2.15 cell growth was approximately phenocopied by chemical inhibition of ERK1/2. However, inhibition of JNK1/2, p38, or NF-κB activity had no analogous influence on HepG2.2.15 cell growth. Taken together, our data suggest that the effect of TLR4 on HBV-related HCC cell proliferation is at least partially mediated through regulation of ERK1/2 activation. Since HBx has been shown to induce phosphorylation of ERK1/2 and promote the proliferation of liver cells [28], the interaction between TLR4 and HBx may be involved in the regulation of ERK1/2 activation in HCC cells by TLR4.

However, we have to admit that NF-κB and MAPKs are limited research pathways. ERK1/2 may be the concerned signaling, and it shall not be the only one. In the future we will further investigate the possible pathways of TLR4 involved in HCC, and its relationship with HBx. Although HepG2.2.15 cell is just a single HBV-related cell line, its mRNA and protein expression level is highest among all 5 cell lines. Besides that, HepG2.2.15 cell carries HBV. Therefore, HepG2.2.15 cell has sufficient characteristics and representativeness for the research. We are conscious that if more cell lines are investigated, then as a result more sufficient evidence can certainly be provided to our experiement. We wish to achieve this in the upcoming experiments. Additionally, to further improve the accuracy of the experiments, we plan to upregulate TLR4 as positive treatment in the future.

## Conclusions

Our data show that TLR4 is required for the growth and survival of HBV-related HCC cells, which is mediated, at least partially, through ERK1/2 signaling. The interaction between TLR4 and HBx may be involved in the regulation of ERK1/2 activation by TLR4. TLR4 may thus represent a therapeutic target for HCC, especially HBV-related HCC.

## Abbreviations

BSA: bovine serum albumin
CCK8: cell counting kit-8
DAPI: 4′,6-diamidino −2-phenylindole
DMEM: Dulbecco’s modified Eagle’s medium
HBV: hepatitis B virus
HBx: HBV X protein
HCC: hepatocellular carcinoma
LPS: lipopolysaccharide
MAPKs: mitogen-activated protein kinases
NSCLC: non-small cell lung cancer
PDTC: pyrrolidine dithiocarbamate
PMSF: phenylmethylsulfonyl fluoride
qPCR: Quantitative Real-Time Polymerase Chain Reaction
siRNAs: small interfering RNAs
TLR4: toll-like receptor 4
